# Advancing family wellbeing through a Massive Online Open Intervention: the LightBEAM program protocol for randomized waitlist control trial {1a}

**DOI:** 10.1186/s40359-025-03326-3

**Published:** 2026-02-02

**Authors:** Madissen Sitka, Kaeley M. Simpson, Allyson Paton, Anna MacKinnon, Tracie O. Afifi, Emily E. Cameron, Tasmia Hai, Ashley Stewart-Tufescu, Andrea Gonzalez, Elaine Toombs, Jo Ann Unger, Ryan Giuliano, Aislin Mushquash, Mandy Archibald, Lianne Tomfohr-Madsen, Leslie E. Roos

**Affiliations:** 1 Department of Psychology, University of Manitoba, 190 Dysart Road, Winnipeg, MB R3T 2N2 Canada; 2 Department of Pediatrics and Child Health, University of Manitoba, Winnipeg, MB Canada; 3 Faculty of Medicine, Université de Montréal, Montréal, QC Canada; 4 Faculty of Education, University of British Columbia, Vancouver, BC Canada; 5 Faculty of Nursing, University of Manitoba, Winnipeg, MB Canada; 6 Faculty of Education, Simon Fraser University, Burnaby, BC Canada; 7 Psychiatry and Behavioral Neuroscience, McMaster University, Hamilton, ON Canada; 8 Clinical Health Psychology, University of Manitoba, Winnipeg, MB Canada; 9 Faculty of Social Work, University of Manitoba, Winnipeg, MB Canada; 10 Department of Psychology, Lakehead University, Thunder Bay, ON Canada; 11 Rady Faculty of Community Health Sciences, University of Manitoba, Winnipeg, MB Canada

**Keywords:** Massive Online Open Intervention (MOOI), Parenting, Mental health

## Abstract

While early childhood remains a formative period for healthy child development, it is simultaneously a time when parents experience increased mental health challenges. Such increased challenges (e.g., heightened parental stress, anxiety, depression, etc.) can lead to a variety of adverse child outcomes, including increased risk of externalizing and internalizing disorders. Despite the well-documented effects of parental mental health on child development, Canadian families face a plethora of barriers to accessing services in a timely manner, and as such, low-barrier, accessible mental health supports are needed. In an effort to increase available services to Canadian families, the current study translates the BEAM program (Building Emotional Awareness and Mental Health; thebeamprogram.com), a previously established online parenting and mental health app, into LightBEAM a massive open online intervention (MOOI). By translating an efficacious intervention into a MOOI format, we hope to increase accessibility, scalability, and reach of the program in an effort to better support Canadian families. To investigate the efficacy of translating BEAM into LightBEAM, a pilot waitlist control design will be utilized. Canadian families who are parenting a child between the ages of 1.5 and 8 years old and who are experiencing elevated symptoms of parenting stress, anxiety, depression, and/or anger will be recruited. When moving through the intervention arm of the trial, participants will have access to an app that houses 12 weeks of parenting and mental health videos, a group forum, and weekly exercises to reinforce key skills learned. A combination of Multilevel modeling and mediation models will be utilized to assess changes in participants' elevated mental health scores across the 12-week program and the 6-month follow-up period. Additionally, post-program focus groups will be conducted to further explore the feasibility and acceptability of the program. The translation of BEAM into LightBEAM, a national MOOI, has the potential to increase the scalability of this intervention to ensure that Canadian families have access to timely mental health and parenting supports. Results will be used to inform future iterations of the program. This trial was retrospectively registered with ClinicalTrial.gov NCT07026838 on June 10th, 2025{4}. Protocol Version 1, August 2025 {2}.

**Supplementary Information:**

The online version contains supplementary material available at 10.1186/s40359-025-03326-3.

## Background and rationale {9a,b}

Early childhood is a formative period, with the parent–child relationship playing a critical role in subsequent cognitive and social development (e.g., Landry et al. [18], Lugo-Gil & Tamis-LeMond [20]). When parents of young children experience elevated distress (e.g., depression, anxiety, and anger), it can disrupt the ability to form or maintain healthy, dyadic relationships. This puts children at risk for negative outcomes, including difficult temperaments, altered cognitive development, insecure attachment and socioemotional risks [10, 17, 19, 22, 23, 26, 32, 34, 37]. Furthermore, the Intergenerational Transmission of Stress model, which has been used extensively to assess child developmental trajectories (see Bowers & Yehuda [7], Hammen et al. [11]), posits that children exposed to elevated parental distress experience increased levels of stress themselves. This leads to an increased risk of experiencing their own mental health challenges, linked to both genetic and environmental influences [11]. The risks of negative child developmental outcomes are positively correlated with parental distress severity (e.g., Mensah & Kiernan [23], Shonkoff & Fisher [32]). Furthermore, interventions designed to improve parental mental health have also contributed to improvements in child functioning [9]. By ensuring Canadian parents receive adequate mental health supports, we can effectively disrupt the intergenerational transmission and adverse impact of mental health concerns within the family [27]. Despite the well documented impacts of untreated parental distress on child development [17, 22], Canadian families continue to face a multitude of barriers to accessing effective and timely mental health resources (e.g., long wait times, limited free, community or public health resources) [24]. These barriers to accessing parental mental health supports put Canadian children at greater risk for cognitive and socioemotional challenges. Lack of access to timely treatment can worsen the severity of symptoms among families, increasing their distress and challenges over time. Low-barrier interventions that provide timely support can help mitigate these effects and reduce the strain on existing healthcare services.

In response to the growing need for accessible family mental health support, an app-based program entitled BEAM (Building Emotional Awareness and Mental Health; thebeamprogram.com) was launched in 2020. Through several pilots and randomized controlled trials (See Joyce et al. [13], MacKinnon et al. [21], Xie et al. [38], Xie et al. [39]), the BEAM program has offered a combination of parenting and mental health supports including (1) expert-led videos and related activities (e.g., exercises to reinforce skills from videos) (2) online closed group forums (3) symptom monitoring and (4) trial specific online group telehealth sessions led by clinical psychologists or parent-peer coaching. The program has seen positive changes in parental mental health, including improvements in parent anxiety, anger, and sleep, as well as reduction of harsh and negative parent–child interactions [21]. To support families across Canada, we propose the translation of BEAM into LightBEAM, a Massive Online Open Intervention (MOOI) delivered through an app-based platform. By translating BEAM into a MOOI format, we hope to be able to reach and support more families nationwide as the current BEAM trials have been available to only select provinces and limited by clinician availability.

Massive Online Open Intervention (MOOIs) deliver open access, evidenced-based behavioral interventions online through websites or apps [25]. Participants have full autonomy to access these self-directed interventions, which increases intervention reach and scalability by negating the limitations associated with individualized services (e.g., number of available clinicians in each community; Kazdin [15], Muñoz et al. [25]). Furthermore, the MOOI design can address common barriers to accessing services (e.g., cost associated with individualized services; Muñoz et al. [25]). Despite the potential strengths of the MOOI design, a one-size fits all approach to online mental health supports may not be appropriate for all individuals and the lack of direct clinical contact and individualized services leaves room for risk factors to be missed. Given both the strengths and limitations surrounding the MOOI format, more research is needed to explore the success of such programs in the context of mental health service delivery.

In response to the need for more empirical evidence for Massive Online Open Intervention mental health interventions, we are translating BEAM, an evidenced-based parenting and mental health app intervention into LightBEAM, a MOOI app. Our aim is to create an accessible mental health support for Canadian families. While several different MOOI programs exist, many are designed to support broader self-management health behaviors (e.g., smoking cessation programs), with few specifically focused on the translation of mental health and parenting interventions. The potential use of LightBEAM has a broad reach, providing support to families in remote areas with limited in-person services, offering interim assistance while they await individualized care, and serving as a self-referral program. By supporting parent mental health, the LightBEAM program may help foster healthy child and family development, thereby disrupting the negative impact of parental distress on healthy child development.

### Objectives {10}

There are three main aims of the proposed study. The first aim is to assess the feasibility and acceptability of the LightBEAM program. Metrics to examine feasibility will include recruitment and retention rates, participant satisfaction, and an exploration of unmet needs. As a way to explore feasibility, we will utilize qualitative focus groups to examine ways in which the program can be improved to better support Canadian families. Aim two is to examine the efficacy of LightBEAM at improving family outcomes when compared to a waitlist control condition. Primary outcomes for parents include mental health, distress, and parenting quality. Secondary outcomes for children include child well-being and socioemotional development. Aim three is to identify groups associated with the greatest program engagement and improvements. This will promote equity and be used to inform future programming.

### Trial design {12}

Using a two-arm clinical design, participants will be randomized in a 1:1 allocation to be placed in either the 12-week LightBEAM program or the waitlist control. After the 12-week LightBEAM program has been completed by the intervention condition, participants in the waitlist control group will have one month to complete the “re-baselining questionnaires and online orientation prior to gaining access to the LightBEAM intervention. Participants in the intervention condition will complete post-questionnaires at the same time that the waitlist control completes re-baselining questionnaires. Longitudinal self-report questionnaires will be administered digitally at three timepoints: pre-intervention, post-intervention, and at six months follow-up.

## Methods: participants, interventions and outcomes

### Study setting {13}

The study will take place in Canada across all ten provinces and three territories. The study coordination team will be located in the center of Canada, in Winnipeg, Manitoba. Participants will be Canadian primary caregivers of children (1.5–8 years old) who are experiencing elevated levels of anger, depression, anxiety, and/or parental stress. Participants will be able to participate in LightBEAM across a variety of settings (e.g., at home, in rural and remote communities, at work etc.) as long as they are able to access the internet.

### Participant identification, recruitment, and consent {20, 32a}

Participants will be recruited from across Canada using a variety of online social media posts (e.g. Instagram, Facebook), radio ads, and through local organizations (e.g., daycares, community centers etc.). Once recruited, participant informed consent will be obtained via REDCap on two separate occasions; prior to completing the eligibility screener and before becoming enrolled in the program (i.e. before completing pre-questionnaires). Participant Canadian residency will be verified through checking their IP addresses. Should we not be able to verify their residency through IP, we will require participants to upload a photo of their Canadian identification (e.g., driver’s license, passport) alongside a photo of themselves. This verification process is designed to ensure only Canadian residents are accessing the LightBEAM program. The study team has successfully used this form of identity verification across other iterations of BEAM.

During the main consent process, all eligible participants will be asked if they would like to invite a co-parent to complete the LightBEAM program alongside them. Here, we define co-parent as the child’s other parent, stepparent, or other primary caregivers (e.g., child’s grandparents, uncle, auntie, etc.). If the main parent wishes to invite a co-parent to the program, they will be asked to enter their co-parent's email address. The co-parent will then complete a main consent form. Once co-parents have consented, they will go through the same identity verification, randomization, and assessment procedures outlined below.

### Eligibility criteria {14a}

Eligible participants must (a) identify as a primary caregiver of any gender to an 18 -to 107-month-old child (e.g., 1.5 - 8 years of age), (b) reside in Canada with access to an internet connection, (c) be aged 18 years or older, (d) be able to understand/read/speak in English, (e) report mild emotional distress across at least one of the following metrics; depression (Patient Health Questionnaire- 9 ≥ 5), anxiety (Generalized Anxiety Disorders Questionnaire ≥ 5) anger (Patient Reported Outcomes Measurement Information System Anger ≥ 14), or parenting stress symptoms (Parenting Stress Index Fourth Edition ≥ 110), and (f) have access to an electronic device. On the screener questionnaire, participants will be asked to contact the study coordinator via email if they do not have access to an electronic device as we will be able to provide a limited number of devices to those who do not have access. Although many parenting programs are open to caregivers who are parenting children under the age of six, LightBEAM includes caregivers who are parenting children up to the age of eight. Such a wide and inclusive age range was intentional to address the many behavioral challenges and delayed diagnosis that children encounter later in childhood, while also considering inclusivity and general lack of programming for parents of children who are older. Participants will be excluded if they are (a) below the age of 18 years, (b) parenting a child outside the designated age range, (c) live outside Canada, (d) have participated in previous iterations of BEAM. Co-parents will be subjected to similar inclusion and exclusion criteria as the main participant, however they will not have to report mild emotional distress. As such to be eligible, co-parents must (a) reside in Canada with access to internet connection, (b) be aged 18 years or older (c) be able to understand, read, and speak English and have access to an electronic device.

### Participant timeline {18}

The LightBEAM program will use a rolling recruitment model whereby eligible participants will be randomized and gain access to the program on the first Monday of each month (e.g., all eligible participants who complete the screener questionnaire during the month of September will be randomized and gain access to the program on the first Monday in October). This rolling recruitment model will continue until the target sample size is reached. While participant timeline in the program will vary within this model, all participants will complete the aforementioned questionnaires in the same order regardless of program start date. Co-parents will engage in the same process described below as the main participant, with the exception that co-parents will not (a) complete the screener questionnaire or (b) complete the Child Behavioral Checklist.

#### Questionnaires {25a, 26}

Participants will complete a series of measures delivered through REDCap, a secure data storage platform maintained by the Centre for Healthcare Innovation in Winnipeg, Manitoba. Questionnaires will be delivered to assess both eligibility and primary and secondary outcomes across both parent and child mental health.

##### Eligibility screener (Main Participants Only)

All primary participants will complete an online eligibility screener to determine if they meet the required inclusion criteria. The eligibility screener consists of several standardized measures to assess depression, anxiety, parenting stress and anger. The Generalized Anxiety Disorder questionnaire (GAD-7; Spitzer et al. [35]) and the Patient Health Questionnaire (PHQ-9 [16]) will be used to examine parental anxiety and depression respectively. These questionnaires ask parents to reflect upon mental health concerns in the past two weeks. Both the GAD-7 (α = 0.92) and the PHQ-9 (α = 0.88) evidence robust internal reliability [16, 35]). The Parenting Stress Index Short Form (PSI-SF) is a 36-item questionnaire that will be used to assess parenting stress. The PSI asks parents to report on a variety of factors including parental distress, parent–child dysfunctional interactions, and difficult child behaviour (e.g., I feel trapped by my responsibilities as a parent; α = 0.87; Abidin et al. [1], Barroso et al. [5]). The Patient Reported Outcomes Measurement Information Symptom [PROMIS] Anger scale will be used to evaluate parenting anger. This ten-item scale asks parents to reflect on experiences of anger in the past seven days (e.g., I was irritated more than people know; α. = 88; Kaman et al. [14]).

##### Pre-intervention, post-intervention and follow-up questionnaires

All eligible participants (i.e., main participants and co-parents) will be asked to complete additional questionnaires that assess other factors known to influence parental mental health such as sleep and self-compassion (i.e. Patient Reported Outcomes Measurement Information Symptom [PROMIS] Sleep Scale [40], The Self-Compassion Short Form Questionnaire [4, 28]). Aspects of the parent–child relationship, child development and family dynamic will also be assessed using a variety of questionnaires (i.e., The Parenting Scale, Irvine et al. [12], Child Behavioral Checklist [3]). Additionally the authors generated an emergency service utilization questionnaire (see Roos et al. [29]) and an adapted version of the National Survey for Children’s Mental Health [8] to assess various sociodemographic, service utilization and health factors related to child and family development. All questionnaires will be completed via REDCap. See Table 1 for a full list of study measures and subsequent timepoints.

**Table 1:** Schedule for questionnaire administration: main participant

LightBEAM Proposed Metrics					
Questionnaire	Eligibility	Pre-Intervention	Re-Baselining	Post-Intervention	Follow-Up
Pre-Screener	X				
Patient Health Questionnaire	X	X	X	X	X
Sociodemographic		X			
Generalized Anxiety Disorders	X	X	X	X	X
Self-Compassion		X	X	X	X
PROMIS Anger	X	X	X	X	X
PROMIS Sleep		X	X	X	X
Parenting Stress Index	X	X	X	X	X
Social Support Effectiveness		X	X	X	X
CBCL		X	X	X	X
Parenting Scale		X	X	X	X
App Usage				X	
Service Utilization		X	X	X	X
Modified NMH		X	X	X	X

*PROMIS* Patient Reported Outcome Measurement Information Systems, *CBCL* Child Behavioral Check List, *NMH* National Survey for Children’s Mental Health, *App Usage* Author Generated App usage questionnaire

##### Re-baselining questionnaires

All participants who are randomized to the waitlist control condition will complete an additional set of questionnaires prior to gaining access to the online LightBEAM content. Participants in the waitlist control condition will have a one-month period to complete these questionnaires.

#### Online orientation session

All eligible participants who complete the informed consent procedure will be asked to attend a mandatory online orientation session (approximately 60 min) before gaining access to the LightBEAM program. Parent coaches, who are trained research personnel who previously completed a different iteration of BEAM, will lead the online orientation sessions. Coaches will help orient participants to the app, answer questions about the program, and monitor the closed group forum within the app. Participants will book this online orientation through Microsoft BOOKINGS where they will be able to select a time slot that works best for their schedule. Participants randomized to the LightBEAM program will attend this orientation session shortly after completing pre-program questionnaires. Participants randomized to the waitlist control condition will attend this orientation session after completing the re-baselining questionnaires.

#### Intervention {15a}

The LightBEAM program is a ‘light touch’ extension of the previous BEAM program, meaning it maintains the core principles and theoretical underpinnings used to create BEAM without the direct clinical involvement (Building Emotional Awareness and Mental Health; see Joyce et al. [13], Simpson et al. [33], Xie et al. [38], Xie et al. [39]). The BEAM program is an app-based program developed to support parent mental health, parenting, and healthy child and family development through supporting parental child bonding, and emotion-focused parenting. The BEAM program has traditionally consisted of weekly activities, online group forums, online group-therapy, symptom tracking questionnaires and expert led videos which draw from transdiagnostic emotion-focused mental health and third wave Cognitive Behavioral Therapy principles. To increase the number of families who can access and benefit from the BEAM program, the content has been translated onto a self-guided app-based platform, LightBEAM. The LightBEAM app is being developed by MindSea, a Canadian based digital health solutions company. The LightBEAM app will employ the same expert lead videos, exercises, tip sheets, a group forum and weekly symptom tracking as BEAM (see Joyce et al. [13], Simpson et al. [33]), however LightBEAM will not involve direct clinician contact (i.e. the group therapy and peer coach model will be removed). By removing direct clinician involvement, we effectively increase the scalability of the original BEAM program, allowing a greater number of Canadian families to benefit and access this innovative mental health support.

Following the same structure as BEAM, the LightBEAM program will consist of 12 weeks of parenting and mental health video content (See Table 2). Participants will be instructed to move through the videos and practice exercises at a self-guided pace. The LightBEAM program will consist of four different components; weekly parenting and mental health videos, weekly symptom tracking, a group forum, and exercises designed to reinforce skills learned through the video content.

**Table 2:** Schedule for questionnaire administration: co-parent

Questionnaire	Pre-Intervention	Re-Baselining	Post Intervention	Follow-Up
Pre-Screener				
Patient Health Questionnaire	X	X	X	X
Sociodemographic	X			
Generalized Anxiety Disorders	X	X	X	X
Self-Compassion	X	X	X	X
PROMIS Anger	X	X	X	X
PROMIS Sleep	X	X	X	X
Parenting Stress Index		X	X	X
Social Support Effectiveness	X	X	X	X
CBCL				
Parenting Scale	X	X	X	X
App Usage			X	
Service Utilization	X	X	X	X
Modified NMH	X	X	X	X

*PROMIS* Patient Reported Outcome Measurement Information Systems, *CBCL* Child Behavioral Check List, *NMH* National Survey for Children’s Mental Health, *App Usage* Author Generated App usage questionnaire

#### Psychoeducational video content

LightBEAM program consist of a combination of mental health and parenting content developed by Dr. Roos and Dr. Tomfohr-Madsen. Emotion focus parenting strategies, parent management training and theoretical attachment orientations were utilized by Dr. Roos to develop the parenting content. Dr. Roos and Dr. Tomfohr-Madsen integrated a combination of third wave cognitive behavioral and transdiagnostic emotion focused mental health principles to develop the mental health content.

##### Weekly symptom tracking

Participants will complete two author-generated quantitative weekly symptom tracking exercises to promote emotional awareness [30]. These exercises ask participants to reflect upon how they have felt in the past week across various wellness domains (i.e., individually, interpersonally, socially) and how they have felt in relation to parenting outcomes (i.e., frustration, confidence, relationship quality). Within the app, participant scores on weekly questionnaires will populate a graph that is visible to both participants and researchers (See Simpson et al. [33]).

##### Weekly exercises

Within the app, participants will be prompted to complete weekly exercises designed to reinforce key skills discussed in the weekly psychoeducational video content. Exercises will be completed digitally within the app and include a series of true and false or multiple-choice questions that reflect key concepts learned from that week's content.

##### Group forum

Participants will gain access to a confidential group forum embedded into the LightBEAM app. This forum will be a place for participants to reflect upon the intervention content, engage in dialogue with other participants, ask questions, and offer support. The forum will be monitored daily by parent coaches, trained research personnel who have previously completed BEAM. The coaches will monitor the forum for inappropriate posts and content (e.g., content related to self-harm or suicidality, posts with identifying information) and will also help stimulate group conversation. Furthermore, this forum will offer a place for subsequent qualitative data analysis to further examine feasibility and efficacy of the program.

#### Focus groups

Utilizing a data driven approach informed by the collected quantitative data on feasibility and acceptability metrics, we will conduct focus groups to further explore feasibility constructs. A purposive sampling procedure will be employed whereby a sub selection of eligible participants who completed LightBEAM and indicated interest in completing a focus group will be asked to participate after they complete their six-month follow-up surveys. Proposed purposive criteria may include focus groups sampled based on gender, immigrant status, program adherence, and ethnicity. By utilizing such purposive criteria, focus groups will also allow authors to explore how the program served specific populations. In total, 2–3 groups of 5–7 participants per purposive criterion will be conducted. Focus groups will take place over Zoom and be approximately 1.5 h long. Research has demonstrated that focus groups conducted via Zoom evidence high participant satisfaction, strong data quality and improved access for participation [2]. Both audio and video will be recorded, however, only audio recordings will be transcribed and analyzed. Audio recordings will be uploaded to Trint for transcription purposes. Confidentiality will be ensured by instructing participants to refrain from mentioning any personal identifiable details during the focus group (e.g., refrain from saying their or others’ name). If any identifying details are said, these will be redacted from the transcripts. As part of signing the consent form, participants will also be asked to respect fellow participants' privacy and refrain from sharing participant information learned within the interview/focus group.

A manifest content analysis approach guided by established frameworks (e.g., Theoretical Framework of Acceptability; Sekhon et al. [31]) will be used to enhance data quality. Several strategies will be employed to code qualitative data. Established coding rules will determine inclusion criteria and guide interpretation of ambiguous data to ensure consistency across coding team. Phrases and sentences that reflect a distinct idea related to feasibility, acceptability, or user experience will be defined as single unit of analysis. Once coding rules are established, transcripts will be double coded to generate initial impressions, and a series of collaborative team meetings will be used to refine candidate codes. The coding scheme will then be applied systematically across transcripts with a lead qualitative investigator reviewing coding for thematic mapping, patterning and comprehensiveness. Recommendations outlined by Begley and Tobin [36] will be utilized to further enhance trustworthiness of qualitative data including credibility, dependability, confirmability, and transferability. Several strategies will be utilized to employee these recommendations. First, during analysis, the coding team will be instructed to engaging in team-based coding and memoing to support credibility. Dependability and confirmability of data will be supported through the use of an audit trial and analytic insights. Assumptions and positionality will be addressed in team meetings to support reflexivity practices in addition to researcher journaling. Finally, transferability will be addressed by providing contextual details on participant demographics and sampling procedures. An example list of focus group questions will appear in Appendix A.

#### Compensation

Participants will be eligible to earn up to $75 CAD for participation. The compensation will be delivered in the following denominations; (a) $20 for post-intervention completion (b) $20 for completing six-month follow-up questionnaires (c) $35 for participating in optional post-intervention focus groups. Compensation will be delivered in the form an e-gift "Everything Card" which is a type of e-gift card that provides recipients with flexibility to use the funds across a wide range of retailers, both online and in-store.

### Criteria for discontinuing or modifying allocated interventions {15b}

Participants will be asked to discontinue treatment if they break the terms of use associated with the LightBEAM app which includes disclosing personal information on the group forum, engaging in inappropriate forum posts, and not respecting the privacy of other participants either on the forum or during the online orientation session. If a participant is asked to discontinue treatment, or if a participant asks to withdraw, we will email the participant and ask if we can use their partial data. If yes, data will be stored in accordance with REB study guidelines. If a participant does not consent, all partial data with the exception of the initial consent form will be destroyed.

### Strategies to improve adherence and intervention protocols {15c, 25b}

A variety of strategies will be used to increase intervention adherence including email, text and telephone reminders, all of which have improved adherence in previous BEAM trials [13, 21, 33]. Furthermore, this RCT will employ an intent to treat analysis, and as such all participants will be reminded and encouraged to complete questionnaires regardless of their level of participation in the program. Additionally, the app itself was designed with user friendly features, engaging graphics and colours and user-tested to help facilitate adherence and engagement.

#### Tracking concomitant care during trials {15d}

Participants will be allowed to receive concomitant care for mental health problems.

Use of additional interventions will be assessed through a service utilization questionnaire and subsequently controlled for in analyses to account for potential confounding effects.

#### Sample size {19}

In total, we plan to recruit a sample of 200–500 Canadians caregivers to participate in the LightBEAM program based on recruitment success over the funding availability period [33]. Sample size was informed by feasibility based on previous BEAM trials (see Simpson et al., [33]).

## Methods: assignment of intervention {21a-b, 22, 24a-c}

### Allocation

Using the built-in randomization functionality within REDCap, participants will be randomly allocated to either LightBEAM or the waitlist control condition.

### Blinding

Study coordinators, participants, data analysts, and the principal investigator will not be blind to the condition of the participants. All randomization will be completed by the LightBEAM study coordinator using the automated REDCap randomization function. A randomization table will be created and uploaded into REDCap by a data analyst at the Canadian Centre for Health Care Innovation.

## Methods: data collection, management and analysis

### Data collection methods and management {25a}

All data storage and collection will occur in accordance with the University of Manitoba research ethics boards standards. Participants will be informed during the consent process that they are free to withdraw at any time. Additionally, the study coordinators and principal investigators may remove a participant from the LightBEAM trial if they violate the forum’s terms of use.

Questionnaire data will be collected on REDCap. Data captured within the LightBEAM app (i.e., participant weekly questionnaire response and forum posts) will be stored on a Firebase Server and data storage located in Montreal, Canada in the Google cloud.

### Statistical methods {27a, b}

Statistical analysis will occur on both IBM Statistical Package for the Social Sciences (SPSS) and Mplus 8.4. Prior to data analysis, data files will be reviewed to ensure participant questionnaire data are in the expected range and scored correctly. Missing data will be address using an intent-to-treat analysis and maximum likelihood estimation.

#### Aim 1

The feasibility of the LightBEAM program will be evaluated through examining a variety of program specific metrics including participant app usage, program attrition, and self-report implementation metrics. See Table 3 for a full list of pre-specified success criteria. Descriptive statistics (e.g., frequencies, means) will be used to examine participant program implementation metrics. Additionally, a series of qualitative questions will evaluate feasibility and acceptability in the focus groups, building off and extending the understanding of the numerical data. A manifest content analysis will be utilized to quantify and analyze qualitative focus group data. Guided by the feasibility and acceptability framework (see Sekhon et al. [31]), this approach will involve determining unit of analysis and coding rules that are applied to focus group transcripts. Next, a coding team will apply the relevant codes and share initial impressions [6]. Candidate codes will be considered and redefined in a iterative processes. Next, the coding framework will be trialed and finally first pass coding will be reviewed for comprehensiveness, patterning, and mapping.

**Table 3:** Overview of LightBEAM weekly content

Week	Mental Health Topic	Parenting Topic
1	Values (Defining Values and Finding Motivation)	Family Values (finding and following your values)
2	Emotions + Function	Loving your whole child
3	Mindful Emotion Awareness	Helping your child name & understand their emotional experiences
4	Understanding your emotions	Emotions in the Moment (Tantrums + Coping Thoughts)
5	Emotional ARC and doing the unexpected	Meeting your child in their emotions ARC
6	Managing Emotions Reactions	Co-regulating DT + TIPP Skills
7	Anticipating Emotions and challenging moments	Pre-teaching and successful routines
8	Cognitive flexibility	Flexibility through transitions
9	Interpersonal effectiveness and clarifying priorities	Clarifying family boundaries and RESET
10	Navigating Conflict	Conflict management and STOP skills
11	Boundary setting to make space for everything else	Memory making with your kiddo
12	Staying well and moving forward	Recipe for wellness

#### Aim 2

All eligible participants must present with at least one elevated symptom of concern across parental anxiety (GAD-7), depression (PHQ-9), anger (PROMIS) and/or parenting stress (PSI). Based on which metric(s) participants screen in as being elevated, an individualized composite primary score will be created whereby we examine whether there is a statistically significant change in elevated symptoms of concern across assessment time points. To examine the efficacy of the LightBEAM program in improving parental outcomes, a multi-level model with random intercepts will be utilized to assess the effect of treatment over time based on conditions. An intent to treat approach will be followed such that all randomized participants are included in analysis, regardless of completion.

Specifically, we will examine pre-to-post-to follow up program changes in primary and secondary outcomes. Such analysis will allow us to examine the efficacy of the LightBEAM program compared to other community resources. Program engagement metrics (e.g., number of videos watched, see Table 4) will also be included as moderators to probe whether participant changes are due to program engagement. This dosage analysis will be an exploratory analysis.

**Table 4:** Aim 1 feasibility and acceptability metrics

	Indicator	Benchmark	Rational
Recruitment Rate	Number of parents recruited per month	Approx. 50 parents	We want at least 50 parents in the forum to ensure maximum engagement
Retention Rate	% of parents with follow up data	> 70% of parents with follow up data	Average retention rate amongst previous MOOI’s is 70%
Forum Use	# of posts and comments made on the forum	> 50% of users engage at least once in the forum (i.e., through posting, responding or interacting with a message)	This is an exploratory outcome. Based on previous work that showed 57% engagement in the BEAM forum
Videos watched	% of videos watched at least 50% of the way through	Participation in > 50% of the LightBEAM Content	Utilizing same benchmark (e.g., 50%) used in previous BEAM pilot studies [13]
Symptom tracking	# of weekly surveys completed	Participation in > 50% of symptom tracking exercises	Based on benchmark used in previous BEAM pilot studies
Acceptability	Self-reported implementation metrics	Score > 4 considered strong implementation	Based on benchmark used in previous BEAM pilot studies

The acceptability aim is assessed using an online questionnaire designed for app based mental health programs. See Appendix 2 for a full list of questions

#### Aim 3

To examine the characteristics of families who benefited most from the LightBEAM program, demographic characteristics and service-related variables will be included as moderators in the same multi-level model discussed in objective 2. Sociodemographic factors (i.e., age, marital status, number and age of children) will also be examined to determine for whom the LightBEAM program is best suited for.

On an exploratory basis, we will use regression to examine the effects of several potential moderators (e.g., child age, severity of child baseline problems, previous caregiver mental health diagnosis, household chaos, gender of caregiver) to understand how various social groups differentially experience the program.

### Exploratory Analyses {27d}

As an exploratory analyses, mediation models will be used to examine if factors within the Intergenerational Transmission of Stress Model (e.g., parenting quality, parenting stress, family and system level factors) are potential mediators of both parent and child well-being.

### Definition of analysis population related to protocol non-adherence and methods to handle missing data {27c}

The LightBEAM program will employ an intent-to-treat analysis using maximum likelihood estimation, whereby all participants, regardless of study completion and adherence, will be included in analysis based on their initial randomization. To account for treatment adherence, de-identified analyses will be conducted using only data from participants who completed the LightBEAM intervention.

## Methods: monitoring

### Composition of the data monitoring committee {28a}

Given the minimal risk associated with this trial design, recruitment, randomization, and data collection procedures will be monitored by the Principal Investigator and study coordinators. A formal data monitoring committee will not be implemented. Additionally, a Canada-wide team of Co-Investigators will be consulted throughout the study design, implementation and data analysis procedures.

### Interim analysis {28b}

After the first 100 participants have completed the LightBEAM program, an interim data analysis will occur. Specifically, graduate students, principal investigators and senior research assistants will conduct preliminary analysis to assess how LightBEAM has succeeded in addressing the three proposed aims. This initial analysis will help the team identify if the translation of BEAM to LightBEAM is effective at supporting parental mental health and parenting. This will allow the team to re-evaluate the study design and implementation if results suggest we are not adequately achieving such effectiveness. De-identified analyses will be shared with larger co-investigators to assess efficacy of LightBEAM.

### Frequency and plans for auditing trial conduct {29}

Our research team has no plans to conduct an internal audit during this trial. The Research Ethics Board at the University of Manitoba may conduct an external audit of this research project.

## Ethics and dissemination

### Research ethics approval {30}

This protocol was approved by the University of Manitoba Research Ethics Board August 2024 [HE2024-0152]. All adverse events will be reported to the University of Manitoba Research Ethics Board, in accordance with their protocols and policies.

### Protocol amendments {31}

All required protocol amendments will be submitted and implemented in accordance with the University of Manitoba’s REB protocols.

### Confidentiality {33}

All efforts will be made to protect participant confidentiality throughout the course of the LightBEAM program in accordance with the University of Manitoba's Research Ethics Board policies. Participant data and identifying information will be stored separately within a password protected SharePoint account maintained by the University of Manitoba. Furthermore, all research personnel will be TCPS and PHIA certified and complete an oath of confidentiality. Additionally, efforts will be made to protect confidentiality during the online orientation sessions and within the group forum. Participants will be informed that confidentiality may be breached in the event that imminent danger to self or others is disclosed to researchers, including child maltreatment or in the event of a court subpoena.

### Declaration of interest {28}

The authors associated with this RCT have no interest to declare.

### Access to data {6}

All data will be stored in a secure location accessible by study coordinators and Principal Investigators. Participant level data will not be made available. Summary statistics, statistical code, and questionnaire data will be made available upon reasonable request.

### Ancillary and post-trial care {34}

All participants will receive a list of crisis resources upon completion of the eligibility screener. A provincial-specific list of community resources and counselling services will be provided to participants via email if they are not eligible to participate in the LightBEAM study.

### Dissemination and policy {8,11}

Efforts will be made to disseminate findings broadly to both academic and public audiences through implementation of an integrated knowledge mobilization plan. Specifically, a series of knowledge mobilization presentations geared towards both lay and academic audiences will be used to disseminate findings in tangent with academic articles published in peer review and open access journals. All participants will be asked during the informed consent process if they would like a copy of the results. Additionally, findings will be shared with collaborating agencies, community partners and policy makers through various community facing presentations.

## Discussion

The LightBEAM program is a light touch extension of our previous program BEAM, which evidenced improvement in mental health and parenting outcomes across several large-scale randomized trials (see Joyce et al. [13], MacKinnon et al. [21]). The LightBEAM program has the potential to offer Canadian families evidenced-based mental health support by translating a previously established intervention into an easily accessible app based MOOI. By creating a program that is free, user-friendly, and widely accessible across Canada, we negate common barriers faced by Canadian families when accessing mental health resources (i.e., long wait times, limited services in rural communities), thereby mitigating the negative downstream impact of untreated parental mental health concerns on child and family development. Findings will provide information on the feasibility, acceptability, and efficacy of the LightBEAM program. Results and participant feedback will be used to inform and improve future iterations of the LightBEAM program. Pending evidence for both the feasibility and acceptability of the LightBEAM program, future aims include the potential for adaptations tailored to fit different cultural, community, and/or diagnostic contexts.

### Trial status

This trial has been registered on ClinicalTrails.gov NCT07026838. Recruitment is set to begin in March 2025. We anticipate that the first 100 participants will be enrolled throughout the spring of 2025, with their 12-week program finishing in July of 2025. Recruitment is anticipated to end by December 2025.

## Supplementary Information

Supplementary Material 1.

## Data Availability

No datasets were generated or analysed during the current study.

## Appendix 1

### Sample LightBEAM focus group questions

1. Can you tell me about your experience accessing the LightBEAM eHealth mental health service? What was your experience with this service?
2. Can you tell me how relevant you felt the parenting content you saw on LightBEAM was? Was the content helpful to you as a parent?
3. Can you tell me how relevant you felt the mental health content you saw on LightBEAM was? Was the content helpful to you as a parent?
4. What were your favorite and least favorite components of LightBEAM
5. What made the program easy to use?
6. Where there any barriers to accessing this service?
7. Were any aspects of the program hard to use? Did any of the program components feel irrelevant?
8. What else would you like to see in the LightBEAM Program?
9. Would you recommend this program to a friend?

## Appendix 2

### LightBEAM acceptability metric

*Response options range from Agree to Disagree on a seven-point Likert scale.*

1. The App was very easy to use
2. It was easy for me to learn to use the app
3. The navigation was consistent when moving between the screens
4. The interface of the app allowed me to use all the functions (such as entering information, responding to reminders, viewing information offered by the app)
5. Whenever I made a mistake using the app, I could recover easily and quickly
6. I like the interface of the app
7. The information in the app was well organized so I could easily find the information needed
8. The app adequately acknowledged and provided information to let me know the progress of my action
9. I feel comfortable using the app in social settings
10. The amount of time involved in using the app has been fitting for me
11. I would use this app again
12. Overall, I am satisfied with this app
13. The app would be useful for my health and well-being
14. The app improved my access to healthcare services
15. The app helped me manage my health effectively
16. This app has all function and capabilities I expected it to have
